# Supplementary material for: Single-cell entropy for accurate estimation of differentiation potency from a cell's transcriptome
Source: Nat Commun. 2017 Jun 1;8:15599. doi: 10.1038/ncomms15599 (PMC5461595; doi:10.1038/ncomms15599)
Supplement: Supplementary Information — Supplementary Figures, Supplementary Tables and Supplementary References [file ncomms15599-s1.pdf]

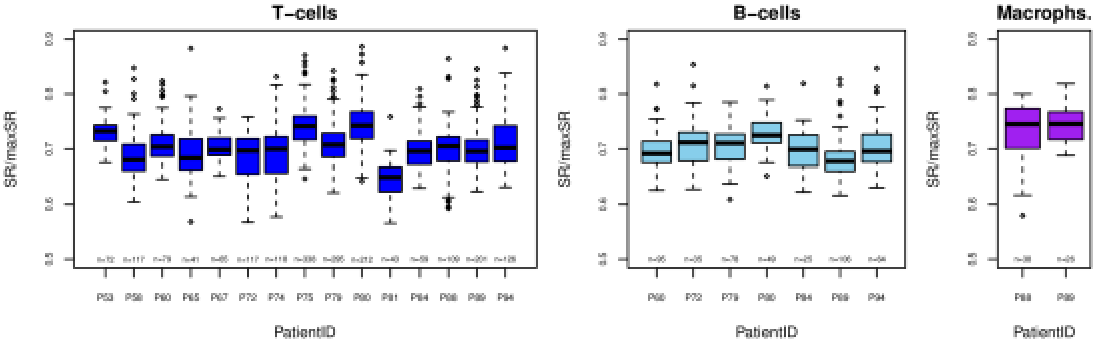

Supplementary Fig.1: Relative stability of entropy values of cell-types across different patients. Boxplots of single-cell normalized entropy values (SR/maxSR) for 3 different cell-types across patients (x-axis). The number of single-cells for each patient is given below each box.

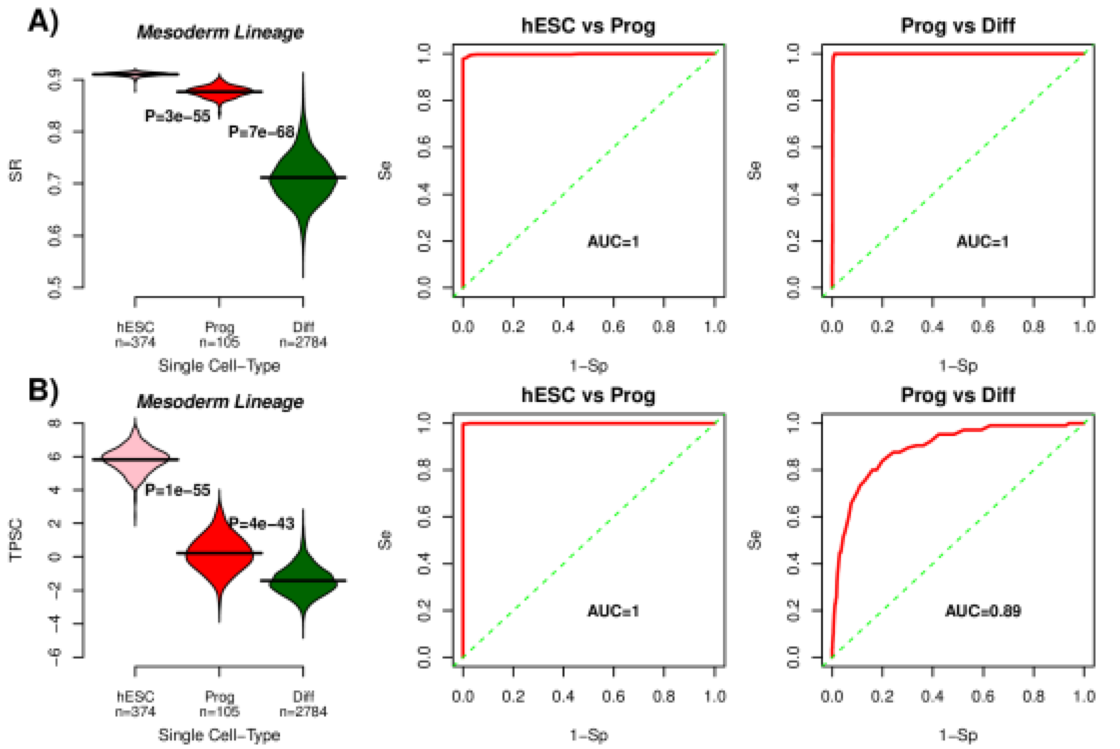

Supplementary Fig.2: Comparison of discriminative power of differentiation potency between signaling entropy (SR) and the t-test pluripotency score (TPSC). Left panels depicts violin plots of the potency scores across single cells representing three different potency states within the mesoderm lineage (hESC: pluripotent, Prog: multipotent mesoderm progenitors, Diff: differentiated endothelial cells within mesoderm lineage, as described in Fig.2). P-values are from a Wilcoxon rank sum test comparing respective potency groups. Right panels depict the ROC curves and AUC values for the corresponding comparisons, as indicated. **A)** for signaling entropy (SR), **B)** for the pluripotency score obtained from the pluripotency expression signature of Palmer et al [1].

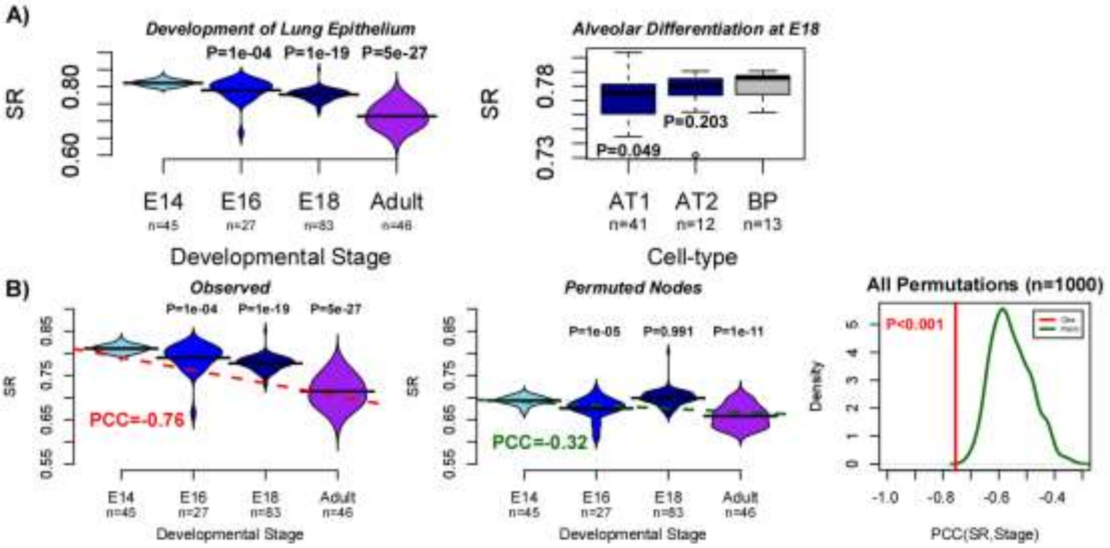

Supplementary Fig.3: The importance of the network in Signaling Entropy. A) Left panel: Signaling entropy (SR, y-axis) as a function of developmental stage in the differentiation of the distal mouse lung epithelium (Treutlein set). Number of single cells measured at each stage is given. Wilcoxon rank sum test P-values between embryonic day 14 (E14) and all other stages are given. **Right panel:** Signaling entropy (SR, y-axis) of alveolar type 1 and 2 cells as well as putative bi-potent progenitors (BP) at E18. Wilcoxon rank sum test P-values between AT1 and BP, and between AT2 and BP are given. **B)** Comparison of the observed Pearson Correlation Coefficient (PCC) between signaling entropy (SR) and developmental stage (left panel) to the corresponding PCC for a random reshuffling of the expression values over the network before SR computation (middle panel). Right panel compares the null distribution of PCC values for a total of 1000 permutations (green curve) to the observed PCC value (red line). No permutation led to a PCC value more extreme than the observed (P<0.001).

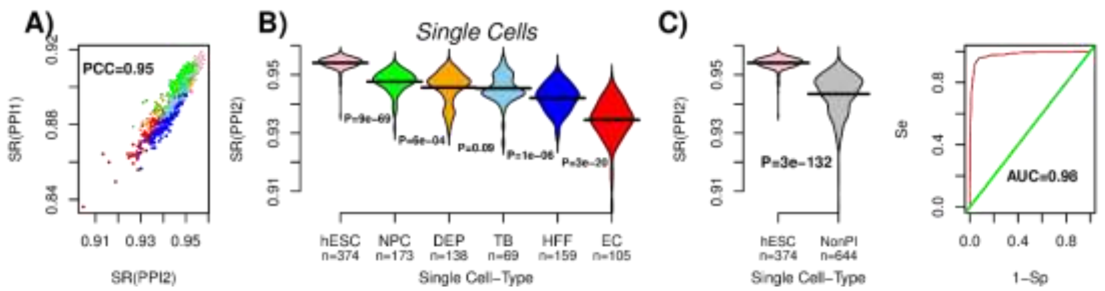

Supplementary Fig.4: Robustness of relative signaling entropy values to choice of PPI network. A) Scatterplot of the signaling entropy for one version of a PPI network (SR(PPI1)) against that computed for another version (SR(PPI2)). Pearson Correlation Coefficient (PCC) is given and estimated over the values for 1018 single cells from Chu et al [2]. Both PPI networks were derived from Pathway Commons using a procedure as described in [3], but differ in the date-stamp of download with PPI1 (number of nodes=8434) derived from the 2012 version of PathwayCommons and PPI2 derived from the 2016 version (number of nodes=11751). Cell-type is indicated by color-see panel-B. **B)** Violin plots of the signaling entropy computed using PPI2 against cell-type (hESC=human embryonic stem cells, NPC=neural progenitor cells, DEP=definite endoderm progenitors, TB=trophoblast cells, HFF=human foreskin fibroblasts, EC=endothelial cells (mesoderm progenitor). Number of single cells in each class is indicated. Wilcoxon rank sum test P-values between each cell-type (ranked in decreasing order of SR) are given. **C)** Left panel: Violin plot comparing the signaling entropy (SR(PPI2)) between the hESCs and all other (non-pluripotent) cells. P-value is from a Wilcoxon rank sum test. Right panel: the associated ROC curve, which includes the AUC value. Observe how the patterns shown in B) and C) are in line with those shown in Fig.2, demonstrating overall robustness of the relative signaling entropy values to the choice of PPI network.

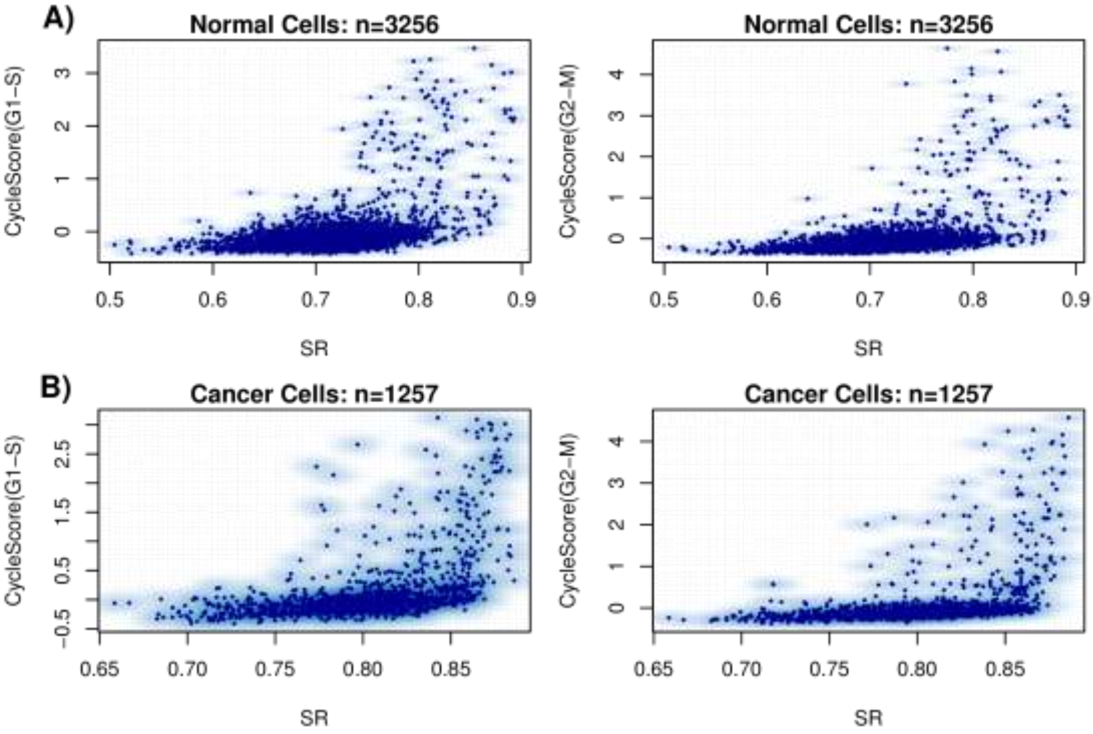

Supplementary Fig.5: Non-linear association between signaling entropy and cell-cycle phase. Smoothed scatterplots of the cell-cycle scores (G1-S for left panels and G2-M for right panels) of single cells versus their signaling entropies (SR) for A) non-malignant cells and B) cancer cells found in the microenvironment of melanomas (scRNA-Seq data from [4]). Number of cells is indicated above plot.

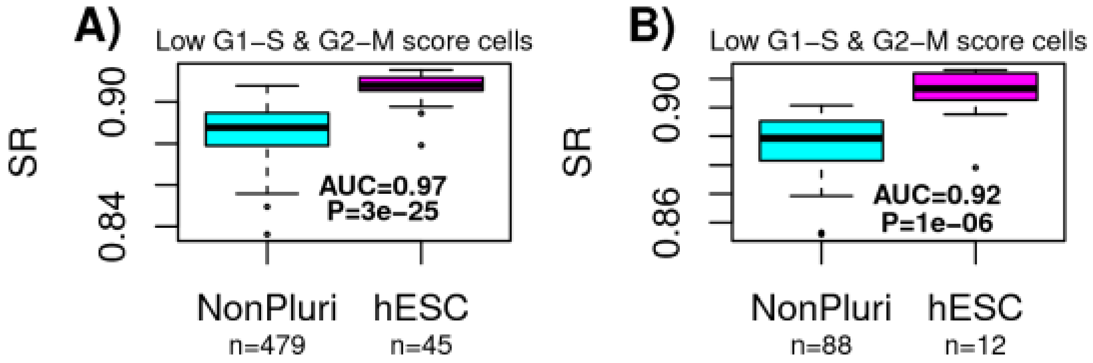

Supplementary Fig.6: Signaling Entropy correlates with differentiation potency, independently of cell-cycle phase, in Chu et al dataset. A) Signaling Entropy (SR) vs cell-type (pluripotent hESCs vs non-pluripotent cells), using only low-cycling cells as determined using a 0.25 threshold for the cell-cycle scores G1-S and G2-M. P-value is from a one-tailed Wilcoxon rank sum test, and the associated AUC classification accuracy statistic is given. **B)** As A), but for low-cycling cells defined as the cells with the lowest 5% cell cycle scores in each cell-type.

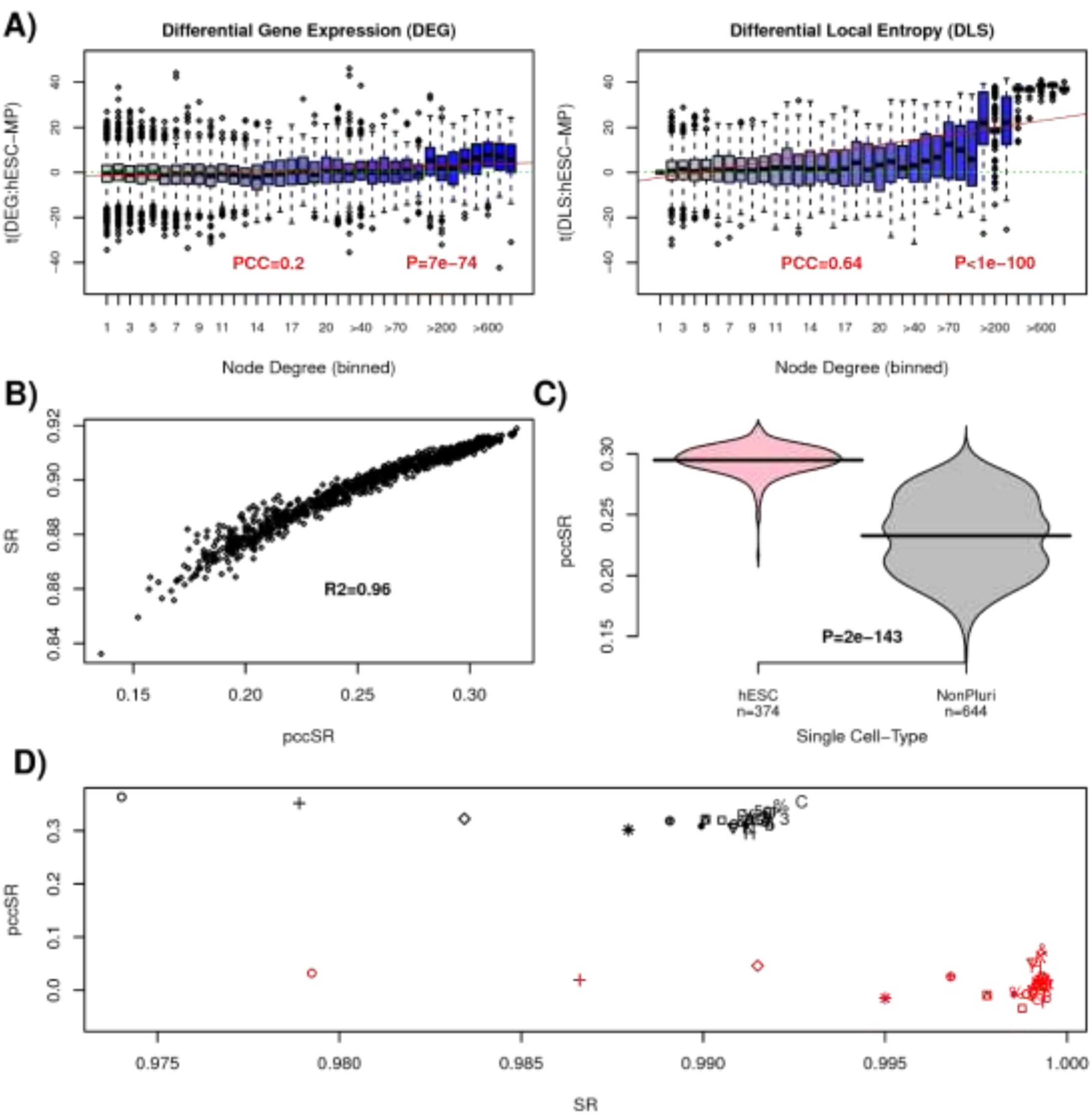

Supplementary Fig.7: Local signaling entropy, Pearson correlation approximation and non-equivalence of Signaling Entropy and correlation approximation. A) Left panel: Boxplot of the t-statistics of differential gene expression (y-axis) against gene/node connectivity (x-axis) with node degrees binned in groups as indicated. Pearson correlation coefficient and associated P-value are given. Red line is the least squares regression. **Right panel:** As left panel, but now for the t-statistics of differential local entropy (y-axis). **B)** Scatterplot of signaling entropy (SR) versus its Pearson correlation approximation (pccSR) for all 1018 single cells of Chu et al dataset. R^2 is given. **C)** pccSR as a discriminator of pluripotent and non-pluripotent cells in Chu et al dataset. Wilcoxon rank sum test P-value is given. **D)** Scatterplot of Signaling Entropy (SR, x-axis) against its Pearson Correlation Approximation (pccSR, y-axis) for 100 simulated networks (labeled by different symbols/letters). For each network there are two data points shown in two colors (red & black), representing different gene expression distributions. As we can see, the black data points correspond to an expression distribution where there is a positive correlation (pccSR~0.3) between expression and connectivity, whereas the red data points correspond to an expression distribution where there is no correlation between expression and connectivity

(pccSR~0). However, for each of the 100 simulated networks, the signaling entropy (SR) is higher for the expression distribution that is uncorrelated with the degree (red points). Each of the 100 simulated networks contained 1010 nodes, with 10 nodes representing hubs, each with a connectivity of 100, with the rest of the 1000 nodes having low-degree (1-5 connections). Importantly, in these simulated networks the hubs are NOT connected to each other. They are only connected via intermediate low-degree nodes. In real PPI networks, hubs are generally connected to other hubs, and in this scenario (as shown in e.g. SuppFig.3C), there is excellent agreement between SR and pccSR.

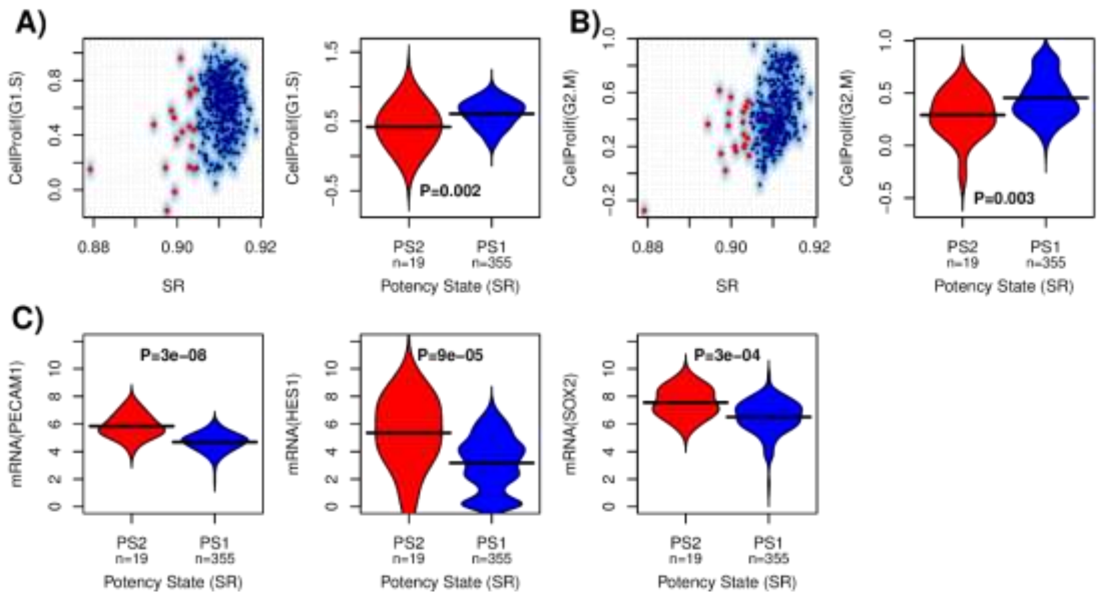

Supplementary Fig.8: Biological characteristics of the non-pluripotent "hESC" subpopulation identified by SCENT. A) Scatterplot of a cell-proliferation index (G1-Synthesis phase) against the signaling entropy rate (SR) for a total of 374 hESCs from Chu et al [2]. Red denotes hESC cells classified by SCENT into a low potency state (PS2), with blue denoting hESCs classified into a high potency state (PS1). Right panel shows the corresponding bean (density) plots, with the P-value derived from a one-tailed Wilcox rank sum test. **B)** As A), but for the cell-proliferation index measuring the G2-M phase of the cell-cycle. **C)** Beanplots of the mRNA expression levels of 1 mesoderm stem-cell marker (PECAM1) and two neural stem cell markers (HES1 and SOX2) in the 374 hESCs, stratified according to their potency state as inferred with SCENT: PS1=highest potency, PS2=low potency. P-values are from a one-tailed Wilcox rank sum test.

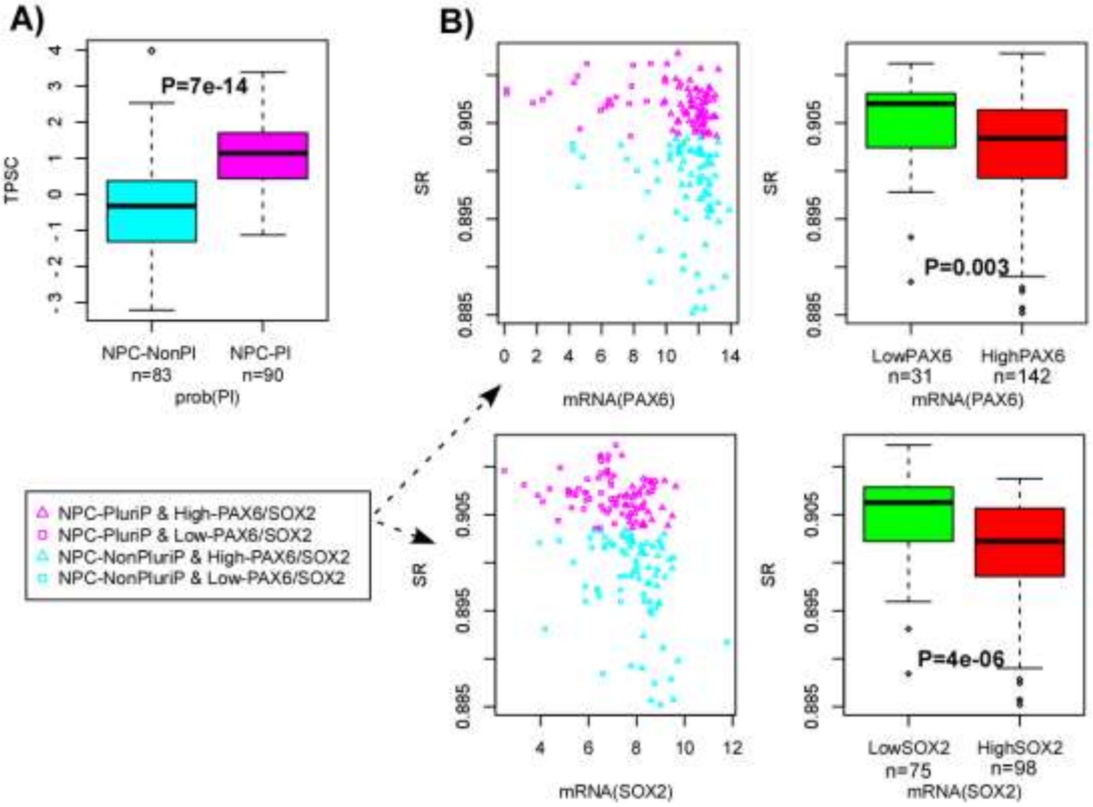

Supplementary Fig.9: Biological significance of the two NPC subpopulation predicted by SCENT. A) Boxplot comparing the t-test based pluripotency score (TPSC) from Palmer et al pluripotency signature in the NPCs from Chu et al [2], stratified according to the predicted SCENT potency state (NonPl=Non-pluripotent(PS2), Pl=Pluripotent(PS1)). **B)** Left panels: scatterplots of signaling entropy (SR) against mRNA expression levels of two neural stem-cell markers (PAX6 and SOX2). Colors and shapes label potency state and expression level group, respectively, as indicated in legend. Low and high expression subgroups of NPCs were inferred using pam-clustering (k=2). Right panels: boxplots of SR vs low and high expression groups with one-tailed Wilcox rank sum test P-values, testing the alternative hypothesis that NPCs with high expression of neural stem cell markers have a lower potency than “NPCs” expressing lower levels of these markers.

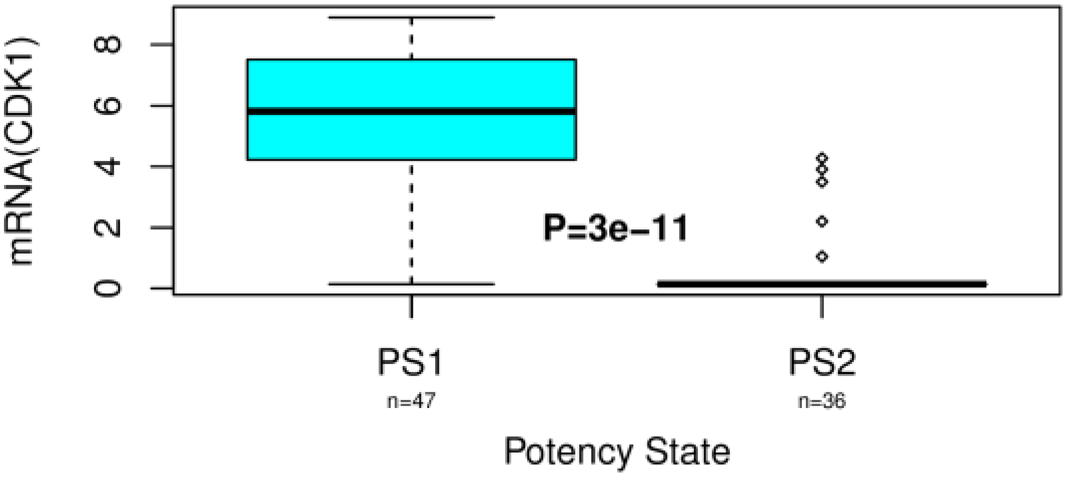

Supplementary Fig.10: Different cycling properties of initiating cells in myoblast differentiation experiment. Boxplot comparing mRNA expression of CDK1 for myoblasts at differentiation induction, stratified according to inferred potency state (PS1=high potency, PS2=medium potency). Number of cells in each potency state is given. P-value is from a two-tailed Wilcoxon rank sum test.

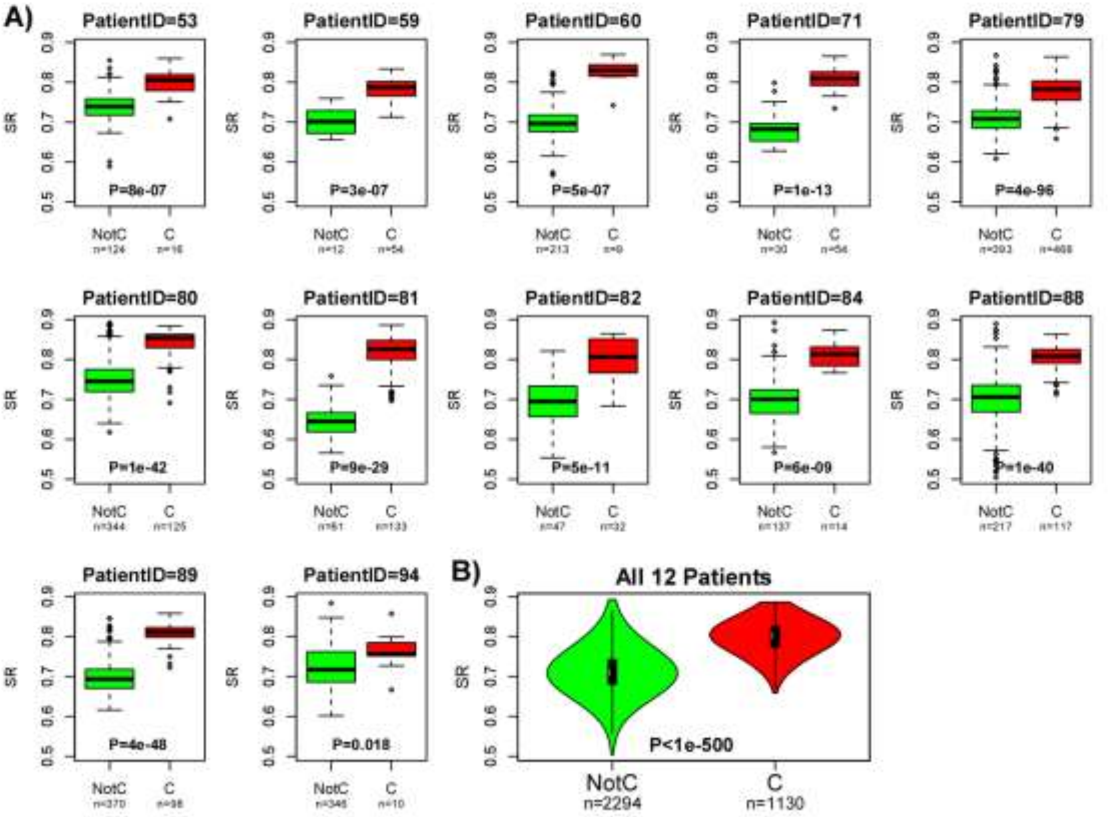

Supplementary Fig.11: Increased signaling entropy in cancer compared to non-malignant cells. A) Boxplots of the signaling entropy (SR) for single melanoma cancer cells (C) compared to non-malignant (NotC) cells for all 12 melanoma patients (patient IDs given above each plot). Numbers of single cells are given below each boxplot. P-value is from a Wilcoxon rank sum test. **B)** As A), but now pooled across all 12 patients.

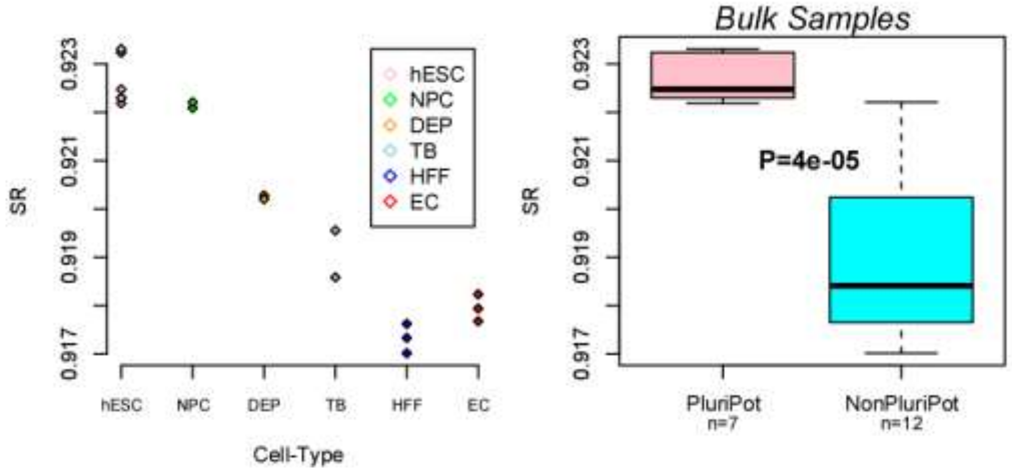

Supplementary Fig.12: Signaling Entropy variation in bulk samples of Chu et al. Signaling entropy of the bulk samples from Chu et al against cell-type, as indicated. Right panel is a boxplot comparing the signaling entropy of 7 hESC bulk samples to those of all other non-pluripotent bulk samples. P-value is from a Wilcoxon rank sum test.

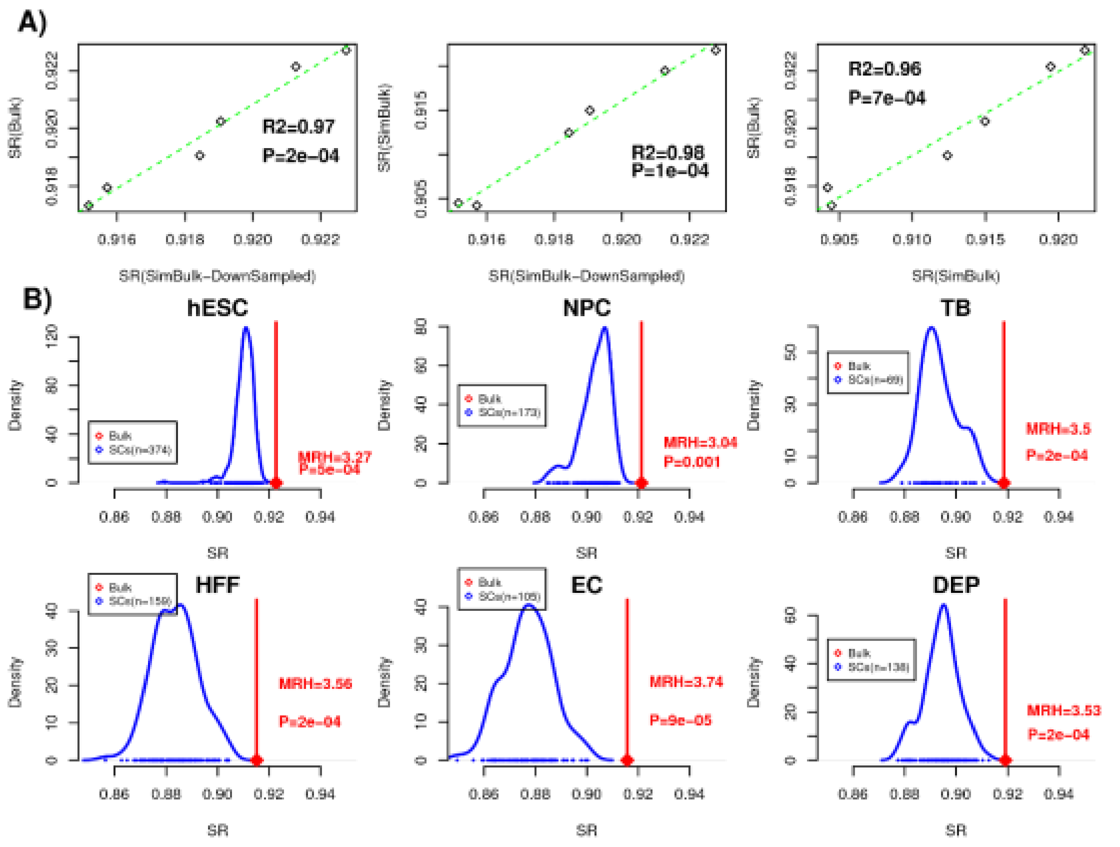

Supplementary Fig.13: Robustness of Signaling Entropy to downsampling and MRH results in Chu et al. A) Scatterplot of the signaling entropies (SR) for the bulk samples ("Bulk") of Chu et al against the corresponding entropies estimated from simulated bulk samples which use scRNA-Seq data. The case where scRNA-Seq data was averaged before computing SR is denoted by "SimBulk", whereas the case where reads from the simulated bulk sample were downsampled to match the average number of reads per single cell is denoted by "SimBulk-DownSampled". R2 values and P-values from a linear regression are given. There are 6 data points, one for each cell-type (hESC, NPC, HFF, TB, DEP, EC). **B)** For each cell-type, each plot compares the distribution of single-cell signaling entropies (SR) (blue) to that of the simulated bulk sample (red line) using downsampling to correct for the larger number of dropouts in single-cell data. The z-statistic (MRH) and associated P-value are given.

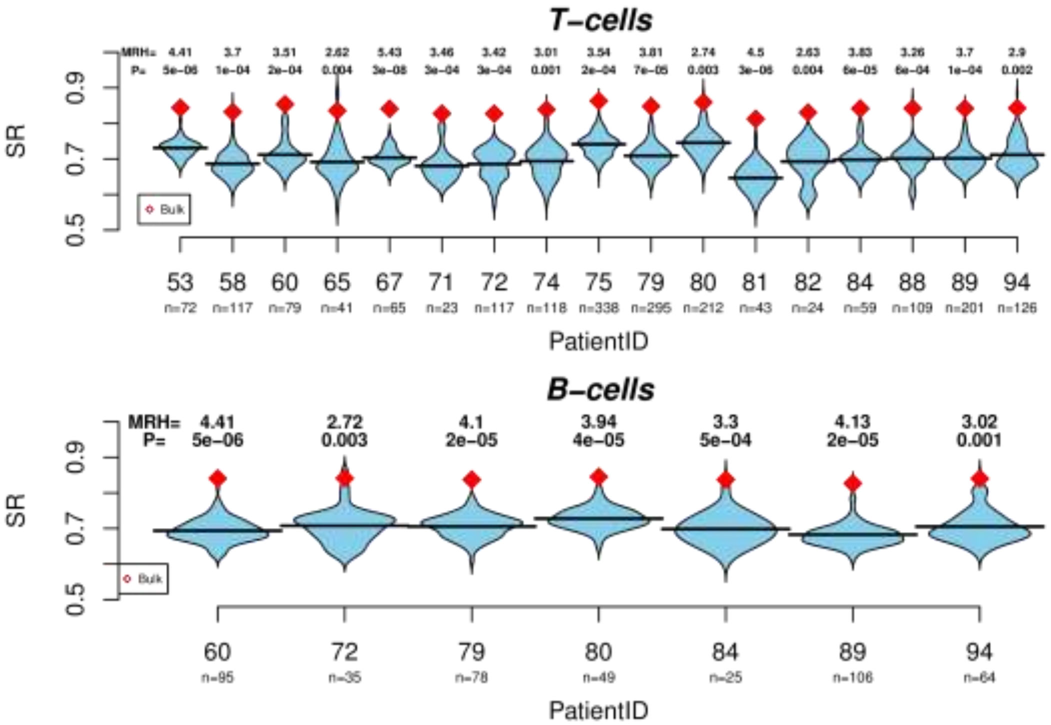

Supplementary Fig.14: Regulated Expression Heterogeneity of T and B-cells in the tumour microenviroment. Beanplots showing the distribution of signaling entropy values (SR) for single T and B-cells across different melanoma patients with at least 20 single cells profiled. Red diamonds show the SR values obtained by averaging the expression profiles of the single cells ("Bulk"). For each patient, we provide the measure of regulated heterogeneity (MRH) and the corresponding P-value, indicating the likelihood that the SR value of the bulk could have arisen from picking at random a single cell from the cell population. Number of single cells for each patient is indicated below beanplot.

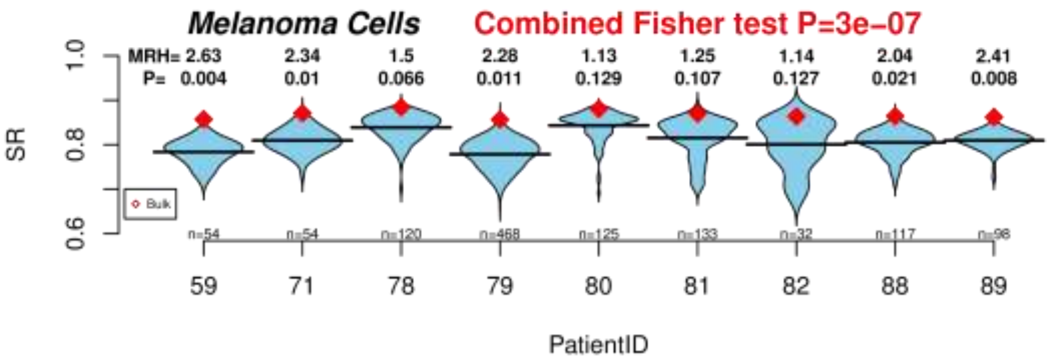

Supplementary Fig.15: Regulated Expression Heterogeneity of Melanoma cells. Beanplots showing the distribution of signaling entropy values (SR) for single melanoma cells across different melanoma patients with at least 20 single cancer cells profiled. Red diamonds show the SR values obtained by averaging the expression profiles of the single cells ("Bulk"). For each patient, we provide the measure of regulated heterogeneity (MRH) and the corresponding P-value, indicating the likelihood that the SR value of the bulk could have arisen from picking at random a single cell from the cell population. We also provide the P-value from a Combined Fisher-test. Number of single cells for each patient is indicated below beanplot.

**scRNA-Seq Dataset**	**Platform**	**Number Single Cells**	**Cell-Types**	**Accession**
Melanoma [4]	Illum.NS500	4645	Melanoma Cancer, T-cells, B-cell, NK-cells, CAFs, EndoC	GSE72056
AML [5]	IllumHiSeq2000	96	Acute Myeloid Leukemia Cells	GSE83533
Chu et al [2]	IllumHiSeq2500	1776	hESC, NPC, DEP, HFF, TB, EndoC	GSE75748
hESC [6]	IllumHiSeq2000	124	Oocyte, Zygote, Early & Late Blastocyst, hESC	GSE36552
Trapnell et al [7]	IllumHiSeq2000	372	Myoblasts->Skeletal Muscle Cells	GSE52529
CTC-PCa [8]	AB5500xlGA	169	Prostate cancer CTCs	GSE67980
Treutlein [9]	IllumHiSeq2000 & IllumMiSeq	201	Mouse lung epithelial cells during development	GSE52583

Supplementary Table 1: Main scRNA-Seq datasets used. Table columns label the name of the dataset used here, the NGS sequencing platform used to generate the data, the number of single cell samples, the type of cells profiled and the public accession number to the data.

**SR ~ Potency + cell-cycle score (G1-S) + cell-cycle score (G2-M)**	**t-stat**	**P**
Potency	9.86	**6e-22**
Cycle Score (G1-S)	14.16	1e-41
Cycle Score (G2-M)	18.56	2e-66
**SR ~ Potency** (only cells with cell-cycle scores <0.25)	**t-stat**	**P**
Potency	13.00	**1e-33**
**SR ~ potency** (only 5% lowest cycling cells in each cell-type)	**t-stat**	**P**
Potency	5.82	**7e-8**

Supplementary Table 2: Signaling Entropy (SR) correlates with differentiation potency independently of cell-cycle phase in Chu et al dataset. Table summarizes the results of three different regression analyses, where SR is correlated to potency (as defined by cell-type, with hESCs (value=1) defining pluripotent cells (n=374) and all other cell-types (value=0) from Chu et al (n=644) being non-pluripotent). In the first regression, the cell-cycle scores for G1-S and G2-M were added as covariates. In the other two analyses, cells have been selected either as those with cell-cycle scores less than 0.25 (45 hESCs vs 479 other cell-types), or as cells within the lowest 25% quantile of both cell-cycle scores in each cell-type (12 hESCs vs 88 other cell-types). Columns label the t-statistic and P-value from the linear regressions.

ENRICHED BIOLOGICAL TERM	OR	AdjP	Example Genes
HETEROGENEOUS_NUCLEAR RIBONUCLEOPROTEIN_COMPLEX	>300	8E-12	HNRNPUL1 HNRNPF HNRNPA2B1 PTBP1 HNRNPDL HNRNPL
28S_RIBOSOMAL SUBUNIT_MITOCHONDRIAL	244.7	1E-22	MRPS16 MRPS17 MRPS2 DAP3 MRPS10 MRPS11 MRPS14 MRPS15
RIBOSOMAL_SUBUNIT	144.9	4E-13	MRPS35 MRPL51 MRPS16 MRPS28 MRPL41 MRPS15 MRPS22
CDC5L_COMPLEX	136.4	5E-22	BCAS2 BZW1 CDC5L CWC15 DYNC1H1 GCN1 HSPA8 ILF2 PLRG1
39S_RIBOSOMAL SUBUNIT_MITOCHONDRIAL	100.9	9E-29	MRPL1 MRPL10 MRPL11 MRPL12 MRPL13 MRPL14 MRPL15 MRPL20
BASC_COMPLEX	92.6	5E-09	ATM BLM MLH1 MRE11A MSH2 MSH6 NBN RAD50 RFC1 RFC2 RFC4
BRM_SIN3A_HDAC_COMPLEX	92.6	5E-09	ACTL6A ARID1A HDAC2 PRMT5 SIN3A SMARCA2 SMARCB1 SMARCD1
C_COMPLEX_SPLICEOSOME	28.5	6E-32	AQR CDC5L CWC15 DDX23 DDX41 DHX8 EFTUD2 EIF4A3
MRNA_SPLICING	18.7	7E-37	ALYREF HNRNPR SRRM1 SF3B4 SF3A1 PRPF8 SF3A3 HNRNPA0 CLP1 SF3B2 NUDT21
G2_M_CHECKPOINTS	18.5	1E-16	CDK2 DBF4 CHEK1 ORC6 ORC3 HUS1 MCM2 MCM3 MCM5 ATM
SPLICEOSOME	17.0	4E-15	U2AF2 SNRNP200 HNRNPM DDX23 U2AF1 API5 SNRPA1 EFTUD2
RNA_SPLICING	11.8	4E-19	PRPF4B HNRNPF CWC15 SNRNP200 SYNCRIP ZNF638 NONO SRSF6
EMBRYONIC_STEM_CELL	5.7	6E-34	EIF4A1 NUP107 RFC3 AURKB PABPC1 FBL MRPS30 MSH2 BUB1 GLDC RUVBL2

Supplementary Table 3: Top-ranked enriched biological terms associated with increased local signaling entropy in pluripotent cells. Table lists a selection of X biological terms from the Molecular Signatures Database [10], which were most strongly enriched among the top-ranked genes, with genes ranked according to increased local entropy in hESCs compared to non-pluripotent cells using the scRNA-Seq data from Chu et al [2]. Columns in table give the name of the term, the odds ratio (OR) of enrichment, the adjusted P-value from a Fisher's one-tailed exact test and a few examples of genes present in the overlap. Biological terms were selected according to a significant P-value after adjustment for multiple testing ($P<1e-7$) and demanding an OR > 5. For the full table of 137 highly enriched terms and genes see Supplementary Table 4 (a separate file).

REFERENCES

1. Palmer NP, Schmid PR, Berger B, Kohane IS: **A gene expression profile of stem cell pluripotentiality and differentiation is conserved across diverse solid and hematopoietic cancers.** *Genome Biol* 2012, **13:**R71.
2. Chu LF, Leng N, Zhang J, Hou Z, Mamott D, Vereide DT, Choi J, Kendziorski C, Stewart R, Thomson JA: **Single-cell RNA-seq reveals novel regulators of human embryonic stem cell differentiation to definitive endoderm.** *Genome Biol* 2016, **17:**173.
3. Banerji CR, Miranda-Saavedra D, Severini S, Widschwendter M, Enver T, Zhou JX, Teschendorff AE: **Cellular network entropy as the energy potential in Waddington's differentiation landscape.** *Sci Rep* 2013, **3:**3039.
4. Tirosh I, Izar B, Prakadan SM, Wadsworth MH, 2nd, Treacy D, Trombetta JJ, Rotem A, Rodman C, Lian C, Murphy G, et al: **Dissecting the multicellular ecosystem of metastatic melanoma by single-cell RNA-seq.** *Science* 2016, **352:**189-196.
5. Li S, Garrett-Bakelman FE, Chung SS, Sanders MA, Hricik T, Rapaport F, Patel J, Dillon R, Vijay P, Brown AL, et al: **Distinct evolution and dynamics of epigenetic and genetic heterogeneity in acute myeloid leukemia.** *Nat Med* 2016, **22:**792-799.
6. Yan L, Yang M, Guo H, Yang L, Wu J, Li R, Liu P, Lian Y, Zheng X, Yan J, et al: **Single-cell RNA-Seq profiling of human preimplantation embryos and embryonic stem cells.** *Nat Struct Mol Biol* 2013, **20:**1131-1139.
7. Trapnell C, Cacchiarelli D, Grimsby J, Pokharel P, Li S, Morse M, Lennon NJ, Livak KJ, Mikkelsen TS, Rinn JL: **The dynamics and regulators of cell fate decisions are revealed by pseudotemporal ordering of single cells.** *Nat Biotechnol* 2014, **32:**381-386.
8. Miyamoto DT, Zheng Y, Wittner BS, Lee RJ, Zhu H, Broderick KT, Desai R, Fox DB, Brannigan BW, Trautwein J, et al: **RNA-Seq of single prostate CTCs implicates noncanonical Wnt signaling in antiandrogen resistance.** *Science* 2015, **349:**1351-1356.
9. Treutlein B, Brownfield DG, Wu AR, Neff NF, Mantalas GL, Espinoza FH, Desai TJ, Krasnow MA, Quake SR: **Reconstructing lineage hierarchies of the distal lung epithelium using single-cell RNA-seq.** *Nature* 2014, **509:**371-375.
10. Subramanian A, Tamayo P, Mootha VK, Mukherjee S, Ebert BL, Gillette MA, Paulovich A, Pomeroy SL, Golub TR, Lander ES, Mesirov JP: **Gene set enrichment analysis: a knowledge-based approach for interpreting genome-wide expression profiles.** *Proc Natl Acad Sci U S A* 2005, **102:**15545-15550.
